# Sabina

**Published:** 1888-04

**Authors:** W. J. Hunter Emory


### SABINA.

In post-partum and other emergency uterine hemorrhages Sabina is often indicated. The sphere of usefulness of Sabina seems confined to abortions at or about the third month. This is its keynote symptom, the flow is bright red, partly fluid and partly clotted, accelerated by bearing-down pains in abdomen and groins, and pain from sacrum to pubis.

Extreme aggravation by music.

Not likely to be of service in post-partum hemorrhage.

W. J. HUNTER EMORY.

